# Health-care utilization and expenditures among patients with comorbid bronchiectasis and chronic obstructive pulmonary disease in US clinical practice

**DOI:** 10.1177/1479973119839961

**Published:** 2019-04-08

**Authors:** Frederic Douglas Seifer, Gary Hansen, Derek Weycker

**Affiliations:** 1 St. Lawrence Health System, Potsdam, NY, USA; 2 RespirTech, St. Paul, MN, USA; 3 Policy Analysis Inc. (PAI), Brookline, MA, USA

**Keywords:** Bronchiectasis, pulmonary disease, chronic obstructive, costs and cost analysis, health expenditures, economics

## Abstract

Recent research suggests that bronchiectasis (BE) may be more common than previously believed and that comorbid chronic obstructive pulmonary disease (COPD) is widespread in this patient population. Little is known about the economic burden among patients with BE, and less is known about the burden among those with comorbid BE + COPD. A retrospective matched-cohort design and data from a US health-care claims repository were employed. From the source population comprising adults who had comprehensive medical/drug benefits for ≥1 day in 2013 (i.e. the referent year) and evidence of BE and/or COPD at any time from 2009 to 2013, patients with BE + COPD were age/sex-matched (1:1:1) to patients with BE only and patients with COPD only. For each matched subgroup, annualized levels of respiratory-related and all-cause health-care utilization and expenditures in 2013 were summarized. Source population included 679,679 patients; among those with BE (*n* = 31,027), 50% had comorbid COPD. Mean (95% CI) annual levels of respiratory-related utilization and expenditures among matched patients with BE + COPD (*n* = 11,685) were higher by 2.4–3.5 times versus patients with BE only and 2.0–2.5 times versus patients with COPD only: hospitalizations, 0.39 (0.37–0.41) versus 0.11 (0.09–0.12) and 0.16 (0.14–0.17); ambulatory encounters, 16.5 (16.1–16.9) versus 6.8 (6.6–7.0) and 8.2 (7.9–8.4); and total expenditures, US$15,685 (14,693–16,678) versus US$5605 (5059–6150) and US$6262 (5655–6868). Respiratory-related utilization and expenditures are high among patients with BE or COPD receiving medical care in US clinical practice and are especially high among those with comorbid BE + COPD receiving medical care, emphasizing the importance of identifying and treating this unique patient population. Funding for this research was provided by RespirTech to Policy Analysis Inc. (PAI).

## Introduction

Non-cystic fibrosis (CF) bronchiectasis (BE) is a pulmonary disorder characterized pathologically by permanent bronchial dilatation and severe bronchial inflammation and clinically by chronic productive cough, hypersecretion of mucus, and recurrent infectious exacerbations.^1^ Once thought to be an orphan disease,^2,3^ BE has become the focus of extensive recent research.^4^ Recent estimates of prevalence have found BE to be far more common than previously thought, with between 340,000 and 522,000 adults receiving treatment for the condition in the year 2013, and an annual growth rate of 8%.^5^ There is growing recognition that BE, either by itself or combined with chronic obstructive pulmonary disease (COPD), represents a growing burden on the US health-care system, which has raised calls for increased surveillance in primary care.^6–8^ The prevalence of COPD within BE is reported to range from 26% to 69%,^9^ raising the possibility of a “COPD-bronchiectasis overlap syndrome.”^10^

Despite this attention, little is known about the economic burden of patients with BE and less is known about the economic burden of comorbid BE + COPD. In an early study using data from a private health-care claims repository, Weycker and colleagues reported that annual total health-care expenditures among BE patients exceeded US$13,000 (2001 US dollars), or roughly twice that of patients without the disorder.^11^ In a subsequent study, Seitz et al. reported median inpatient expenditures to be US$7827 among BE patients enrolled in the traditional fee-for-service Medicare program.^12^ In a large German population, Ringshausen et al. found COPD and BE to be commonly comorbid, with a substantial hospitalization rate in each subgroup.^13^ Joish et al. conducted two studies using a commercial claims database, finding that the total mean cost per patient was substantially higher for BE patients than for case-matched controls (US$35,718 vs. US$26,868)^14^ and that the annual incremental cost associated with BE was also higher.^15^ None of these studies, however, attempted to explicate the added burden of comorbid COPD and BE relative to either of these conditions separately.

Patients with BE and COPD represent a challenging cohort in regards to the management of acute exacerbations of their comorbid disease as the treatment for an exacerbation of either disease alone is different than the management of an exacerbation of both. While the use of antibiotics for COPD in the absence of purulent sputum is controversial,^16^ treatment of colonizing pathogens is foundational to the treatment of BE.^17^ Typically, an individual with comorbid BE and COPD will experience an exacerbation of both conditions: worsened obstruction and bronchospasms for COPD and the presence of purulent sputum for BE. This situation requires a treatment strategy that addresses both aspects of their exacerbation. If the comorbid BE is not recognized, the management of the COPD-related exacerbation may be compromised resulting in a suboptimal response to treatment, a prolonged hospitalization, and a greater risk for readmission following hospital discharge.^17^

While there are obvious clinical reasons to identify BE patients within a larger COPD population,^18–20^ it is also necessary to identify health economic motivations for clinics and payors to allocate resources toward additional surveillance within these populations. The need exists for an up-to-date comprehensive estimate of the economic burden of BE and the relative increased burden of combined COPD and BE.

## Methods

### Study design and data source

This study employed a retrospective matched-cohort design and deidentified data spanning 2009–2013 from the Truven Health Analytics MarketScan® Commercial Claims and Encounters (CCAE) and Medicare Supplemental and Coordination of Benefits (MDCR) databases (hereinafter, the “MarketScan Database”). The MarketScan Database is a large repository that comprises medical (i.e. facility and professional service) and outpatient pharmacy claims from a large number of participating private US health plans. A detailed description of the data source may be found in the online supplement.

### Source and study populations

The source population comprised all persons who were aged ≥18 years in 2013 (i.e. the referent year), had comprehensive medical/drug benefits for ≥1 day in 2013, and had evidence of BE and/or COPD from 2009 to 2013. From the source population, the subgroup of patients who had comorbid BE + COPD were age- and sex-matched (1:1:1, without replacement) to patients who had BE only and patients who had COPD only, respectively, and all matched patients were included in the study population. Patients who had evidence of CF were excluded.

The presence of BE was ascertained based on: ≥2 ambulatory encounters with a corresponding diagnosis (ICD-9-CM 494.x) and dates of service ≥30 days apart; one ambulatory encounter with a BE diagnosis, and computed tomography (CT) scan of the thorax (CPT-4 71250, 71260, 71270) within 60 days prior to the encounter; or ≥1 hospitalization with a principal or secondary diagnosis of BE. The presence of COPD was ascertained based on ≥2 ambulatory encounters with a corresponding diagnosis (ICD-9-CM 491.xx, 492.x, 496) and dates of service ≥30 days apart, or ≥1 hospitalization with a principal or secondary diagnosis of COPD. Our case-ascertainment algorithms for BE and COPD are largely consistent with those employed in prior research.^5,11,21,22^

### Study measures

Levels of health-care utilization and expenditures were tallied on the basis of paid medical and outpatient pharmacy claims with dates of service between January 1, 2013 and December 31, 2013 (i.e. during the referent year) and included respiratory-related and all-cause acute-care hospitalizations, acute-care hospital days, ambulatory encounters (overall and by care setting (e.g. physician office, emergency department, hospital outpatient)), and outpatient pharmacotherapy. Respiratory-related hospitalizations were identified based on acute-care inpatient facility claims with a principal diagnosis code for diseases of the respiratory system plus cough, abnormalities of breathing, fever, and viral infections not otherwise specified (ICD-9-CM: 460–519, 079, 786.0, 786.1–786.4, 786.7–786.9).^23^ Respiratory-related ambulatory encounters were identified based on outpatient facility and professional-service claims (e.g. for care provided in a physician’s office, hospital outpatient department, or emergency department) with corresponding codes in any position. Respiratory-related outpatient pharmacotherapy included antibiotics, bronchodilators, and corticosteroids and were identified using National Drug Codes. Health-care expenditures were based on amounts paid by plans and patients for services rendered by providers.

### Data analyses

Demographic and clinical characteristics of patients with BE only, COPD only, and BE + COPD were described including age, sex, geographic region of residence, presence of selected acute and chronic comorbidities, and the use of selected pulmonary therapies during the 1-year period prior to the referent year. Levels of health-care utilization and expenditures during the referent year (2013) were summarized for each subgroup using frequencies and means, and corresponding 95% confidence intervals (CIs); 95% CIs were generated using techniques of non-parametric bootstrapping. Expenditures were expressed in 2013 US dollars. Because not all patients contributed a full year of data (e.g. due to disenrollment during the referent year), study measures were adjusted for differential follow-up (i.e. utilization and expenditures were expressed in terms of levels per patient-year).

## Results

The source population included 679,679 patients who were aged ≥18 years in 2013, had ≥1 day of medical/drug benefits in 2013, and had evidence of BE only (*n* = 15,573), COPD only (*n* = 648,652), or BE + COPD (*n* = 15,454) from 2009 to 2013. Among the BE + COPD subgroup, 11,685 patients were matched (1:1:1, on age and sex) to the BE only subgroup and COPD only subgroup, respectively (Table 1). Mean (SD) age of matched subjects was 69 (13) years, 64% were aged ≥65 years, and 68% were female. The prevalence of acute and chronic comorbidities was generally highest among patients with BE + COPD versus those with BE only or COPD only, including: acute bronchitis, 67% versus 38% and 47%; lung disease (other than BE or COPD), 45% versus 16% and 19%; and post-inflammatory pulmonary fibrosis, 18% versus 8% and 4%. Mean (SD) duration of follow-up during 2013 was 343 (66) days for patients with BE only, 336 (75) days for patients with COPD only, and 337 (74) days for patients with BE + COPD.

**Table 1.** Demographic characteristics and clinical profile of patients with BE only, COPD only, and BE + COPD in US clinical practice.

	BE only	COPD only	BE + COPD
	(*N* = 11,685)	(*N* = 11,685)	(*N* = 11,685)
Patient characteristics			
Age (years)			
Mean (SD)	69 (12.7)	69 (12.7)	69 (12.7)
Median	70	70	70
Age group, years, *n* (%)			
18–34	124 (1.1)	124 (1.1)	124 (1.1)
35–44	251 (2.1)	251 (2.1)	251 (2.1)
45–54	979 (8.4)	979 (8.4)	979 (8.4)
55–64	2908 (24.9)	2908 (24.9)	2908 (24.9)
65–74	3055 (26.1)	3055 (26.1)	3055 (26.1)
≥75	4368 (37.4)	4368 (37.4)	4368 (37.4)
Gender, *n* (%)			
Female	7921 (67.8)	7921 (67.8)	7921 (67.8)
Male	3764 (32.2)	3764 (32.2)	3764 (32.2)
Geographic region, *n* (%)			
Midwest	2342 (20.0)	3888 (33.3)	3117 (26.7)
South	3195 (27.3)	3279 (28.1)	3436 (29.4)
Northeast	2600 (22.3)	2231 (19.1)	2317 (19.8)
West	3288 (28.1)	1980 (16.9)	2514 (21.5)
Unknown	260 (2.2)	307 (2.6)	301 (2.6)
Clinical profile			
Comorbidities, *n* (%)			
Acute bronchitis	4472 (38.3)	5456 (46.7)	7856 (67.2)
Cardiovascular disease	3157 (27.0)	5771 (49.4)	5730 (49.0)
Diabetes	1780 (15.2)	3418 (29.3)	2894 (24.8)
Genetic and related disorders^a^	875 (7.5)	53 (0.5)	1269 (10.9)
Inflammatory bowel disease	216 (1.8)	190 (1.6)	269 (2.3)
Liver disease	434 (3.7)	598 (5.1)	678 (5.8)
Lung disease (other than BE and COPD)	1899 (16.3)	2262 (19.4)	5288 (45.3)
Lung malignancies	207 (1.8)	567 (4.9)	624 (5.3)
Post-inflammatory pulmonary fibrosis	929 (8.0)	407 (3.5)	2112 (18.1)
Pulmonary nontuberculosis mycobacterial disease	906 (7.8)	24 (0.2)	1008 (8.6)
Rheumatoid disease	695 (5.9)	486 (4.2)	1018 (8.7)
Evidence of use of, *n* (%)			
High frequency chest wall oscillation air-pulse generator system	209 (1.8)	5 (0.0)	636 (5.4)
Electric/pneumatic percussor	2 (0.0)	0 (0.0)	13 (0.1)
Oscillatory positive expiratory pressure device	279 (2.4)	13 (0.1)	369 (3.2)
Respiratory suction pump	89 (0.8)	111 (0.9)	167 (1.4)
Cough stimulating device	4 (0.0)	3 (0.0)	10 (0.1)
Nebulizer compressor	1387 (11.9)	2225 (19.0)	4530 (38.8)
Bronchoscopy	2372 (20.3)	662 (5.7)	3394 (29.0)
Supplemental oxygen	1562 (13.4)	2952 (25.3)	5008 (42.9)

BE: bronchiectasis; COPD: chronic obstructive pulmonary disease.

^a^Situs inversus, common variable immunodeficiency, IgG deficiency, allergic bronchopulmonary aspergillosis, and congenital BE.

**Figure 1.**
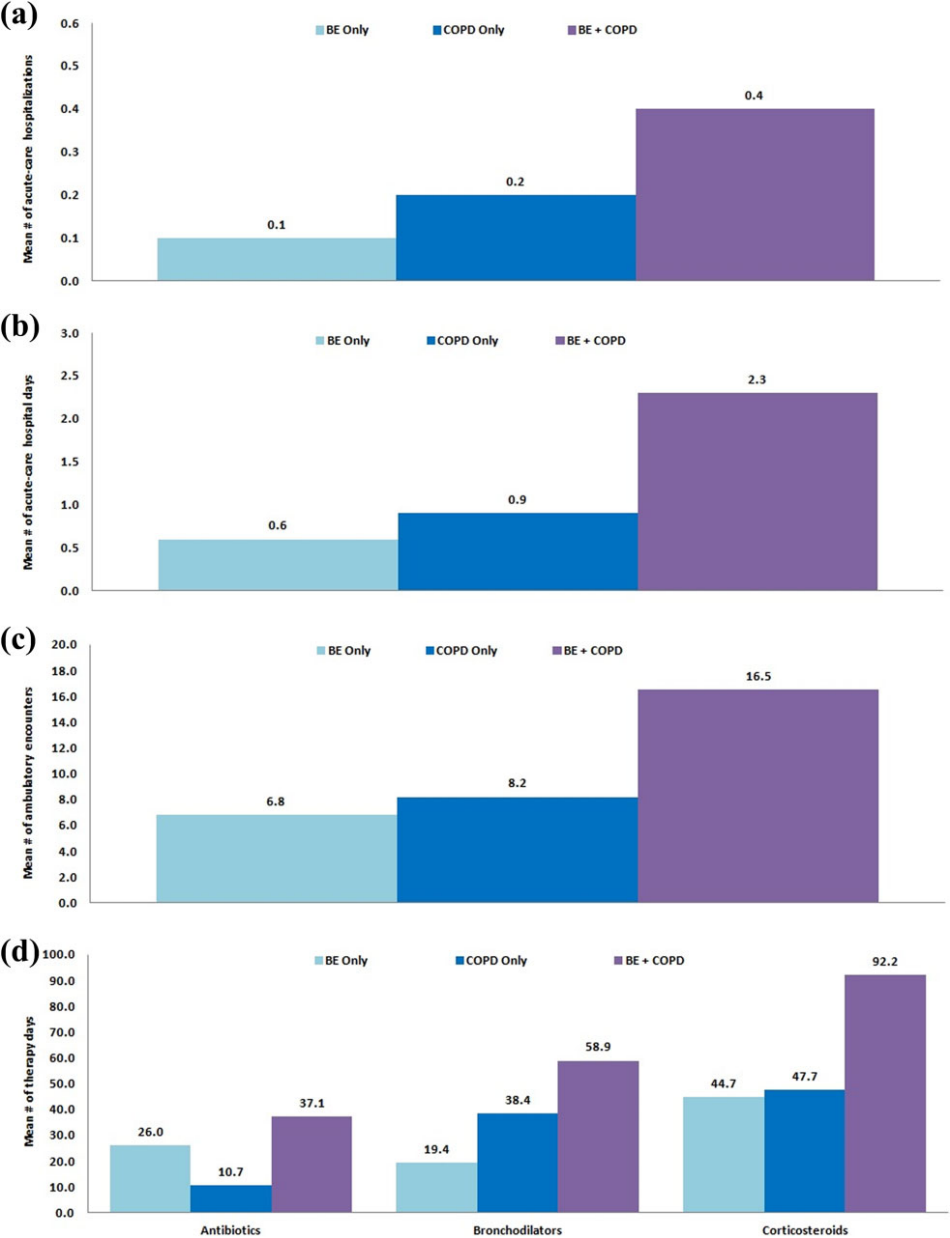
Mean annualized levels of respiratory-related health-care utilization in 2013 among patients with BE only, COPD only, and BE + COPD. (a) Acute-care hospitalizations, (b) acute-care hospital days, (c) ambulatory encounters (any place of service), and (d) prescription medications (outpatient). BE: bronchiectasis; COPD: chronic obstructive pulmonary disease.

**Figure 2.**
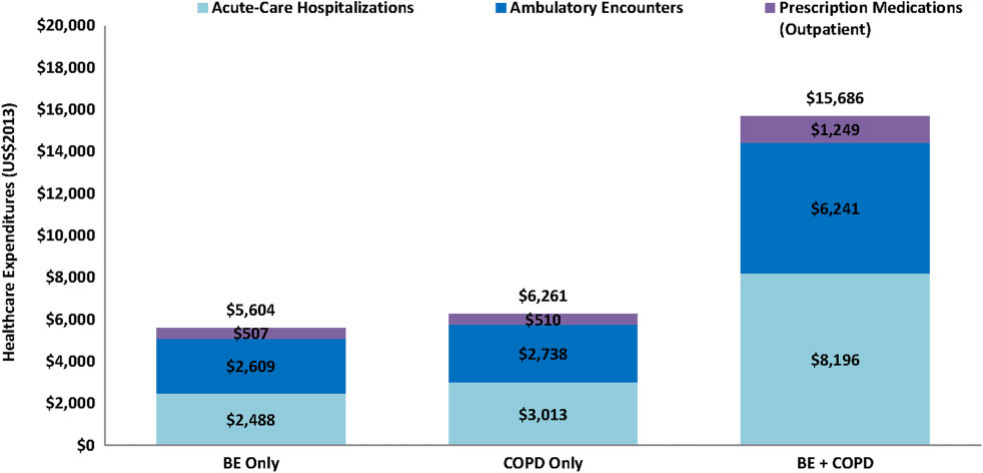
Mean annualized respiratory-related health-care expenditures in 2013 among patients with BE only, COPO only, and BE + COPD. BE: bronchiectasis; COPD: chronic obstructive pulmonary disease.

Mean annualized levels of respiratory-related health-care utilization and expenditure during the referent year were systematically higher among patients with comorbid BE + COPD, exceeding the sum of mean levels for all major categories (except for pharmacotherapy) among patients with either of these conditions separately (Figures 1 to 2). Among patients with BE + COPD, mean (95% CI) number of acute-care hospitalizations was 0.39 (0.37–0.41) versus 0.11 (0.09–0.12) among patients with BE only and 0.16 (0.14–0.17) among patients with COPD only. Mean number of ambulatory encounters (irrespective of care setting) among each of these subgroups was 16.5 (16.1–16.9), 6.8 (6.6–7.0), and 8.2 (7.9–8.4), 40–60% of which occurred in a physician’s office (Online Supplement—Table 1). Mean numbers of outpatient prescriptions (and corresponding mean therapy days) for antibiotics, and especially bronchodilators and corticosteroids, were highest among patients with comorbid BE + COPD.

Mean annualized health-care expenditures for respiratory-related reasons totaled US$15,685 (14,693–16,678) for patients with BE + COPD versus US$5605 (5059–6150) for patients with BE only and US$6262 (5655–6868) for patients with COPD only. Acute-care hospitalizations accounted for 44–52% of total health-care expenditures across subgroups, with most of the remainder (40–47%) accounted by ambulatory encounters. Across major categories of respiratory-related health-care utilization and expenditures (excluding pharmacotherapy), encounters/costs among patients with comorbid BE + COPD were 2.4–3.5 times higher versus patients with BE only and 2.0–2.5 times higher versus patients with COPD only. Mean annualized healthcare expenditures for any reason totaled US$44,212 (42,437-45,987) for patients with BE + COPD, versus US$26,047 (24,740-27,353) for patients with BE only and US$30,567 (29,208-31,926) for patients with COPD only.

While levels of health-care utilization and expenditures for respiratory-related care (excluding pharmacotherapy) represented 20–30% of all-cause care among patients with BE only and COPD only, the percentage among patients with BE + COPD ranged from 30–46%. Accordingly, relative differences in respiratory-related utilization and expenditures were greater than those for the all-cause measures. Results among the unmatched (i.e. full) subgroups were largely comparable to those based on the matched subsets (Online Supplement—Tables 2 to 3).

## Discussion

While COPD may not be understood as a cause of BE, the association of the two conditions is a strong one,^9^ and there is an emerging body of evidence documenting the negative impact of co-existing BE on clinical outcomes among patients with COPD. In one meta-analysis,^24^ study authors reported that the presence of BE was associated with 2.0 times the risk of exacerbation, 4.1 times the risk of colonization of the lungs, and 2.0 times the risk of death when compared to COPD patients without BE. This phenomenon is explainable in the context of Cole’s “vicious cycle,” a repeated cycle of inflammation, exacerbation, lung damage, and downward decline in patient status.^25^ More recently, Suissa found that, unless interrupted, the natural course of COPD is repeated exacerbations, with each being more severe and coming more frequently than the last one.^26^ Clearly, the clinical burden associated with the management of patients with COPD and unrecognized BE (BE + COPD) calls for more attention. Such patients experience a higher risk for severe airway obstruction, colonization by pathogens, and death^24^; additionally, they often have the burden of lower quality of life.^27^ When BE may be found in about half of patients with moderate, severe, and very severe COPD, health-care providers must aggressively seek comorbid BE among the COPD population in order to mitigate the excess clinical impact.

The findings of this economic evaluation extend those from the earlier clinical outcomes studies, by translating elevated risks of clinical outcomes among patients with comorbid BE + COPD into increased economic costs. While the results from this study and other existing evidence suggest that COPD and BE impose major burdens on the US health-care system, the results of this study also suggest that the patient-level burden imposed is markedly higher when COPD and BE are combined. Specifically, the results of this study indicate that levels of health-care utilization and expenditures among patients with comorbid BE + COPD receiving medical care in US clinical practice exceed the sum of corresponding values for patients with BE alone and patients with COPD alone, respectively, receiving medical care in US clinical practice. While it is unknown from this study, it is reasonable to expect that early intervention in BE + COPD patients has the potential to reduce the frequency of exacerbations and associated costs. Accordingly, actions taken to ameliorate the disease process of BE are likely to have a large clinical as well as economic impact.

A few limitations of our study deserve mention. Our operational definitions for BE and COPD, while utilized in other published studies, have not been formally validated against a “gold standard” and thus their accuracy is unknown. For example, while our criterion of ≥2 outpatient encounters with diagnoses of BE or COPD is probably a sensitive measure of case-ascertainment, it may not be sufficiently specific. Moreover, although high-resolution computed tomography (HRCT) has been shown to be highly accurate in diagnosing BE—and is currently the gold standard in this use—ICD-9-CM and CPT-4 procedure codes do not distinguish HRCT from CT with lesser degrees of resolution. We assumed, however, that most persons who underwent CT testing received the recommended HRCT and that any upward bias from using the CT criterion would be small. We could not identify patients who may have been diagnosed with BE and/or COPD but did not have a qualifying encounter during the period of interest. Our case-finding criteria undoubtedly excluded some actual cases of BE and COPD, particularly milder ones without multiple encounters, and thus our estimates of mean levels of disease burden may be inflated. We note, however, that any potential upward bias in estimates of disease burden may have been mitigated somewhat by including patients who were diagnosed with BE and/or COPD at any time during 2009–2013 and thus may not have had qualifying encounters during the referent year (i.e. 2013). We also note that, because of this potential bias, principal attention should be paid to the magnitude of differences between patients with BE + COPD versus patients with BE or COPD alone. While smoking status is an important risk factor for COPD and is undoubtedly an important determinant of disease-related burden, information on smoking status cannot be reliably ascertained from health-care claims. Finally, we note that our study employed data from a convenience—albeit large—sample of persons enrolled in private health-insurance programs in the United States. Persons with such insurance may differ systematically from the rest of the US population in terms of their health status and/or health-care experience as well as from persons in other countries, especially among the elderly. Accordingly, caution should be used in generalizing the results of this study to other populations and settings.

## Conclusions

Levels of health-care utilization and expenditures are high among patients with BE or COPD receiving medical care in US clinical practice and are especially high among those with comorbid BE + COPD receiving medical care (exceeding the combined patient-level burden of those with BE only or COPD only), emphasizing the importance of identifying and treating this unique patient population. This study is important as it is the first to evaluate the economic burden of COPD and BE individually, as well as the combined burden of both. The large incremental burden of comorbid BE + COPD among patients receiving medical care in US clinical practice raises several important implications. First, any formal COPD research that does not recognize and control for BE risks confounded results due to a heterogeneous population. Second, clinicians who do not screen for BE in symptomatic patients with COPD risk undertreating a potentially complex and serious condition. Lastly, the large economic burden that these conditions place on the health-care system should be a call-to-action for organizations that promote treatment guidelines.

## Supplemental Material

Supplemental Material, MS_(Supplement)_--_Economic_Burden_of_BrE_in_US_Clinical_Practice_v1 - Health-care utilization and expenditures among patients with comorbid bronchiectasis and chronic obstructive pulmonary disease in US clinical practice Click here for additional data file. Supplemental Material, MS_(Supplement)_--_Economic_Burden_of_BrE_in_US_Clinical_Practice_v1 for Health-care utilization and expenditures among patients with comorbid bronchiectasis and chronic obstructive pulmonary disease in US clinical practice by Frederic Douglas Seifer, Gary Hansen and Derek Weycker in Chronic Respiratory Disease
