# Supplementary material for: Health-care utilization and expenditures among patients with comorbid bronchiectasis and chronic obstructive pulmonary disease in US clinical practice
Source: Chron Respir Dis. 2019 Apr 8;16:1479973119839961. doi: 10.1177/1479973119839961 (PMC6456842; doi:10.1177/1479973119839961)
Supplement: Supplemental Material, MS_(Supplement)_--_Economic_Burden_of_BrE_in_US_Clinical_Practice_v1 - Health-care utilization and expenditures among patients with comorbid bronchiectasis and chronic obstructive pulmonary disease in US clinical practice [file MS_Supplement_--_Economic_Burden_of_BrE_in_US_Clinical_Practice_v1.pdf]

Healthcare Utilization and Expenditures among Patients with Comorbid Bronchiectasis and Chronic Obstructive Pulmonary Disease in US Clinical Practice:

Online Supplement

Study Design and Data Source

This study employed a retrospective design and data from the Truven Health Analytics MarketScan® Commercial Claims and Encounters (CCAE) and Medicare Supplemental and Coordination of Benefits (MDCR) databases (hereinafter, the "MarketScan Database"). The CCAE database contains data on the health insurance claims of employees of large, self-insured corporations and their dependents, along with data from a few commercial health plans. The MDCR database contains enrollment information, Medicare claims, and employer-sponsored health insurance claims of Medicare-eligible retirees, and includes only plans where both the Medicare-paid amounts and the employer-paid amounts are available and evident on the claims. The data extract for this study was limited to insurers that provided healthcare claims information to the MarketScan Database during the entire 5-year period from 2009-2013.

Data available from each facility and professional-service claim include dates and places of service, diagnoses, procedures performed/services rendered, and quantity of services (professional-service claims only). Data available from each outpatient pharmacy claim include the drug (class) dispensed, dispensing date, quantity dispensed, and number of days supplied. Medical and pharmacy claims also include amounts paid (i.e., reimbursed) by health plans as well as by patients to providers for services rendered. Selected demographic and eligibility information (including age, sex, geographic region of residence, dates of plan eligibility) is available for all health plan enrollees. All data can be arrayed to provide a detailed chronology of medical and pharmacy services used by each plan member over time.

The study extract was de-identified prior to its release to study investigators, as set forth in the corresponding Data Use Agreement. The MarketScan Database has been evaluated and certified by an independent third party to be in compliance with the Health Insurance Portability and Accountability Act (HIPAA) of 1996 statistical de-identification standards and to satisfy the conditions set forth in Sections 164.514 (a)-(b)1ii of the HIPAA Privacy Rule regarding the determination and documentation of statistically de-identified data. Use of the study extract for health services research is therefore fully compliant with the HIPAA Privacy Rule.
